# A comparision of oedema detection with diffusion-weighted imaging and T2-STIR imaging in patients with acute myocardial infarction

**DOI:** 10.1186/1532-429X-15-S1-P25

**Published:** 2013-01-30

**Authors:** Anna Kociemba, Magdalena Lanocha, Katarzyna Katulska, Andrzej Siniawski, Magdalena Janus, Malgorzata Pyda

**Affiliations:** 1 I'st Department of Cardiology, University of Medical Sciences, Poznan, Poland

## Background

The diffusion weighted imaging (DWI) is a well known technique in neuroradiology, due to its ability to detect ischemic regions in brain tissue. A recent, rapid development of the magnetic resonance technology and echo planar imaging enabled the application of diffusion imaging in cardiac examinations.

The aim of this study was to compare the diffusion weighted imaging with widely used STIR sequences in the evaluation of edema in patients with acute myocardial infarction (AMI).

## Methods

The study included 71 patients with AMI – 1-7 days after infarction. MR examinations were performed on a 1.5 Tesla scanner with the use of a six-channel phased-array body coil combined with a six-channel spine matrix coil. STIR and DWI sequences were applied before the contrast administration. Both sequences were acquired at the same planes (HLA, VLA and short axis view). DW images with b-value 50 s/mm^2^ was chosen because of the highest signal intensity both in the edema region and healthy myocardium. We have performed both qualitative and quantitative image analysis. The qualitative analysis included the evaluation of the quality of blood suppression, the presence of motion artifacts and the presence of high signal areas. Two contrast to noise ratios (CNR) were calculated. CNR1 was the contrast between edema and healthy myocardium and CNR2 was the contrast between edema and intraventricular blood pool. The area of edema was measured in both STIR and DWI sequences and compared with the infarct size in LGE images in the same slice position.

## Results

The overall image quality was similar in both sequences. The sensitivity was higher on DWI images (83% vs 61%), the specificity was 90% for both sequences. There was no difference between STIR and DWI in overall CNR1 (18.2 ± 7.4 vs 21.4 ± 11.5 respectively) and CNR2 (29.0 ± 14.9 vs 31.2 ± 20.0). CNR1 in STIR images differed depending on the territory of infarction (21.4 ± 7.1 anterior MI, 18.0 ± 8.8 lateral, 15.4 ± 6.3 inferior, p=0,04). The area of edema was higher in DWI images (9.0 ± 4.0cm^2^ vs 8,7 ± 4.4 cm^2^, p=0.0031).

## Conclusions

Our study confirms that DW EPI is a feasible sequence for the myocardial edema imaging and detects high signal areas (edema) more frequently than STIR.

